# Evaluation of Drinks Contribution to Energy Intake in Summer and Winter

**DOI:** 10.3390/nu7053724

**Published:** 2015-05-15

**Authors:** Olga Malisova, Vassiliki Bountziouka, Antonis Zampelas, Maria Kapsokefalou

**Affiliations:** Unit of Human Nutrition, Department of Food Science and Human Nutrition, Agricultural University of Athens, 75 Iera Odos St., Athens 11855, Greece; E-Mails: olgamalisova@yahoo.gr (O.M.); vboun@hua.gr (V.B.); azampelas@aua.gr (A.Z.)

**Keywords:** water balance questionnaire WBQ, winter, summer, hydration, sugar-sweetened drinks, drink

## Abstract

All drinks hydrate and most also provide nutrients and energy. Our objective was to evaluate the contribution of drinks to total energy intake in summer and winter. Data were obtained using the Water Balance Questionnaire (WBQ) from a sample of the general population in Athens, Greece (*n* = 984), 473 individuals (42 ± 18 years) in summer and 511 individuals (38 ± 20 years) in winter stratified by sex and age. The WBQ embeds a semi-quantitative food frequency questionnaire of 58 foods and the Short International Physical Activity Questionnaire. Data were analyzed for the contribution of drinks to total energy intake. In winter, total energy intake was 2082 ± 892 kcal/day; energy intake from drinks was 479 ± 286 kcal/day and energy expenditure 1860 ± 390 kcal/day. In summer, total energy intake was 1890 ± 894 kcal/day, energy intake from drinks 492 ± 499 kcal/day and energy expenditure 1830 ± 491 kcal/day. Energy intake from drinks in summer was higher than in winter (*p* < 0.001) and in men higher than in women in both seasons (*p* < 0.001 in summer, *p* = 0.02 in winter). Coffee, coffee drinks, milk, chocolate milk and alcoholic drinks contributed approximately 75% of energy from drinks. Fruit juice and sugar-sweetened drinks, including soft drinks and fruit juice based drinks, were consumed less frequently contributing up to 25% of drink energy intake. Drinks contribute approximately 1/4 of total energy intake depending on the energy content of the drink and frequency of consumption. Coffee, dairy and alcoholic drinks were the main energy contributors.

## 1. Introduction

Drinks in the categories of tea, coffee, dairy, fruit juice, soft drinks, diet drinks, energy drinks and alcoholic drinks are available in a large and growing number of choices. Variety satisfies flavor preferences, nutritional requirements, and lifestyle needs, and when a variety of beverages are available, it is more likely to reach euhydration [1].

Drinks contribute approximately 25%–50% of water intake [2,3,4], thus they are important for meeting daily water needs and achieving euhydration. Hydration has been associated with the maintenance of normal physical and cognitive functions [5].

However, it has been argued that drinks contribute significantly to the increasing obesity rates worldwide [6,7], mainly because of their energy content [8,9,10]. In some studies, Sugar Sweetened Beverages (SSB) consumption has been associated with weight gain in children and adults [11], while evidence that decreasing SSBs will decrease the risk of obesity and related diseases such as type 2 diabetes has been viewed as compelling [12]. Energy intake from beverages has increased substantially over the past 40 years by approximately 222 calories per person per day [13].

Nevertheless, drinks from the various categories do not contribute equally to energy intake [14]. The energy content of the drink as well as consumption patterns may suggest otherwise, *i.e.*, some caloric drinks may be consumed rarely thus contributing very little to daily energy intake. It has been argued that replacing sweetened caloric drinks with water can help reduce total energy intake in overweight consumers [15]. Nevertheless, consumption of sweetened caloric drinks is a complex issue that may be regarded beyond the caloric contribution and in the context of dietary patterns [16].

Moreover, likewise to food consumption, various factors such as seasonality, physical activity levels and sex may affect the diversity in drink choice and contribution to total energy intake. These factors have not been fully investigated; further knowledge in this area will elucidate the current question of energy intake from dietary sources and from drinks in particular.

In a previous study conducted in Greece, using the Water Balance Questionnaire (WBQ) tool, we found that in 984 adults, 24% of water intake comes from drinks [17]. There is an unequivocal need to further elaborate on these findings, elucidating the contribution of drinks to total energy intake and balance.

The main objectives of this study were to (a) evaluate the contribution of drinks to total energy intake in 984 men and women in Greece in winter and summer; and (b) explore differences in energy intake from drinks due to seasonality and sex.

## 2. Materials and Methods

We exploited an existing database, built by our research group, on drink and food intake for 984 adults [17]. Data were collected using the WBQ tool [3] from July 2010 to February 2012 in the area of Athens, Greece from 473 individuals aged 16–86 (42 ± 18 years) (221 males) in summer and from 511 individuals aged 16–86 (38 ± 20 years) (245 males) in winter, stratified by age and sex according to the Hellenic Statistical Authority [18]. All volunteers were informed on the objectives of the study and the procedures involved and signed an informed consent. The retrieved data were confidential, and the study followed the ethical considerations provided by The Code of Ethics of the World Medical Association (Declaration of Helsinki). The WBQ incorporates a semi-quantitative food frequency questionnaire validated to record energy intake against three-day diaries (*p* < 0.001) [19] and the IPAQ questionnaire [20], which records energy expenditure. Thus, revisiting our database of approximately 1000 men and women in Greece, and reanalyzing data, findings may be expressed on energy balance.

In particular, the WBQ provides: (A) data on the profile of the participant; (B) recording of consumption for the period of the previous month of solid and fluid food, drinking water or drinks from a semi-quantitative food frequency questionnaire (FFQ) of 58 foods validated for estimating energy intake [19] and the extra sugar and honey that is added to drinks; and (C) estimation of physical activity employing the International Physical Activity Questionnaire (IPAQ) [20], which records duration of physical activity at three activity levels (intense, moderate, mild exercise) or sedentary conditions. The IPAQ has been validated in the past as a cross-national instrument for recording physical activity [20,21,22] and to estimate energy expenditure.

The energy and water content of recorded foods and drinks was analyzed using the Diet Analysis Plus (Wadsworth Publishing Co Inc., Belmont, CA, USA), which uses composition data from the USDA National Nutrient Database [23] and selected Greek foods (*i.e.*, mousakas, pastitsio, Greek yoghurt, lentiles beans soups, several Greek pies, souvlaki, stuffed tomatoes) [24]. Based on total energy intake and energy expenditure, energy balance was evaluated as well.

In this study, “drinks” refers to fresh fruit juice or packed fruit juice 100% without sugar content, sugar-sweetened drinks (SSDs) (fruit nectar with sugar, soft drinks with sugar and energy/isotonic drinks), low calorie soft drinks, tea and herbal infusions, milk (all types of milk, chocolate milk and cocoa drink), milkshake and sherbets, coffee and coffee drinks, and alcoholic drinks (beer, wine and all types of alcoholic drinks). “Drink energy intake (DEI)” refers to energy intake from drinks “total energy intake (TEI)” to energy intake from all sources and “energy intake from foods” to energy intake from solid and fluid foods (e.g., soups). The contribution of drinks groups to total energy intake (data from Malisova *et al.* 2013), presented as total water intake (TWI), *i.e.*, water from drinks, drinking water, solid and fluid foods and as drink water intake (DWI), *i.e.*, water from drinks and drinking water.

## 3. Statistical Analysis

Results are presented as mean ± sd for the normally distributed variables and median (P25–P75) for the skewed ones. Normality was tested using the probability–probability plot (P–P plot) and histograms. Differences between men and women regarding the total energy intake, energy from drinks, energy from foods, energy expenditure, energy balance, water intake, water from drinking water and drinks, water from drinks, water from drinking water, water from foods, water loss and water balance were assessed with the independent sample *t*-test for the normally distributed variables and the Mann-Whittney *U*-test for the skewed ones. Similarly, differences between the four quartiles of energy from drinks were assessed with the one-way ANOVA *F*-test for the normally distributed variables and the Kruskal-Wallis *H* Test for the skewed ones. Significance level was set at 5%. Correlations between the contribution of drinks to water intake in relation to energy in winter and summer were tested by Pearson’s or Spearman’s correlation coefficients for normally distributed or skewed variables. All statistical analyses performed using PASW Statistics 18 (SPSS Inc, Chicago, IL, USA).

## 4. Results

Sex, age, self reported weight and height of participants are in Table 1. Energy intake from drink energy intake and foods are presented for men and women in summer and winter (Table 2) in order to identify differences between seasons and sex. Regarding differences between seasons drink energy intake was higher in summer (*p* < 0.001) but total energy intake (*p* < 0.01) and energy from solid foods (*p* < 0.001) were higher in winter. Difference in energy expenditure was not observed between seasons. Men had higher energy from drink energy intake, total energy intake, and energy expenditure than in women in both seasons (Table 2).

**Table 1:** Participant’s BMI, age and sex distribution during summer and winter.

Characteristics	Total, *n*	Female, *n* (%)	Male, *n* (%)
Winter	511	266 (52)	245 (48)
BMI, kg/m^2^	24.6 ± 4.4	23.3 ± 4.1	26.1 ± 4.3
Age, years	36.9 ± 19.5	35.2 ± 19.1	39.9 ± 19.7
Age, years *n* (%)			
<19	95 (19)	54 (20.4)	41 (17)
20–39	173 (34)	88 (33.2)	85 (35.3)
40–64	161 (32)	85 (32.1)	76 (31.5)
>65	77 (15)	38 (14.3)	39 (16.2)
**Characteristics**	**Total, *n***	**Female, *n* (%)**	**Male, *n* (%)**
Summer	473	252 (47)	221 (53)
BMI, kg/m^2^	24.5 ± 3.9	23.3 ± 3.9	25.9 ± 3.7
Age, years	39.9 ± 17.6	38.6 ± 17.2	42.0 ± 18.1
Age, years *n* (%)			
<19	63 (13)	38 (15)	25(11)
20–39	165 (35)	83 (33)	82(36)
40–64	174 (36)	91(36)	83 (36)
>65	78 (16)	39 (16)	39 (17)

Season differences in drink energy intake were observed for all the groups of drinks, except fruit juice. Particularly, milk/chocolate milk coffee/coffee drinks and SSDs contribute more energy in winter. However, in summer alcoholic drinks contribute higher energy than in winter (Table 3).

Sex differences in patterns of drink energy intake were identified. In winter, men had higher total energy intake from SSDs (*p* < 0.001) and alcoholic drinks (*p* < 0.001), while women had higher intake for tea and herbal infusions commonly sweetened with sugar or honey (*p* < 0.001). In summer a similar pattern was observed, men had higher total energy intake from alcoholic drinks (*p* < 0.001) and SSDs (*p* = 0.07) and women had higher intake for tea and herbal infusions (*p* < 0.001) (Table 3).

**Table 2:** Comparison of energy intake from all sources, also itemized as energy from drinks and from foods, energy expenditure estimated from physical activity and energy balance for the total sample (men and women) during winter and summer and energy balance between men and women.

Energy Balance, Intake and Loss (kcal/day)									
kcal/day	Winter	Summer	*p* ^a^	Winter			Summer		
	Men + Women (*n* = 511)	Men + Women (*n* = 473)		Women (*n* = 266)	Men (*n* = 245)	*p* ^b^	Women (*n* = 252)	Men (*n* = 221)	*p* ^c^
Energy Intake	2082 ± 892	1890 ± 894	0.001	2000 ± 655	2172 ± 1087	0.03	1807 ± 794	1978 ± 988	0.04
Energy from drinks	479 ± 286	492 ± 499	<0.001	451 ± 254	508 ± 313	0.02	418 ± 342	573 ± 621	0.001
Energy from foods	1630 ± 750	1398 ± 663	<0.001	1568 ± 552	1702 ± 913	0.04	1389 ± 638	1405 ± 692	0.80
Energy expenditure	1860 ± 390	1830 ± 491	0.19	1670 ± 307	2074 ± 363	<0.001	1569 ± 199	2115 ± 552	<0.001
Energy Balance	346 ± 897	63 ± 982	<0.001	358 ± 683	334 ± 1092	0.75	233 ± 802	130 ± 1114	<0.001
Water Intake (from all sources)	2891 ± 987	3943 ± 1603	<0.001	2774 ± 878	2813 (2383, 3491)	0.04	3658 ± 1377	4120 ± 1330	0.92

Results are presented as mean ± SD for the normally distributed variables and as P50 (P25–P75) for skewed ones. *p*-Values derived through the independent *t*-test for the normally distributed variable and through the Man-Whitney *U*-test for the skewed ones. ^a^ refers to comparisons between summer and winter for the total sample (males and females together); ^b^ refers to comparisons between males and females in winter; ^c^ refers to comparisons between males and females in summer.

**Table 3:** Comparison of energy intake from groups of drinks for the total sample during winter and summer and between men and women.

Energy Intake (kcal/day)									
Drink Group	Total Sample			Winter			Summer		
	Winter (*n* = 511)	Summer (*n* = 473)	*p* ^a^	Women (*n* = 266)	Men (*n* = 245)	*p* ^b^	Women (*n* = 252)	Men (*n* = 221)	*p* ^c^
Fruit juice 100%	14 (0, 44)	14 (0, 68)	0.3	22 (44, 134)	14 (14, 44)	0.95	14 (0, 45)	14 (0, 75)	0.87
Sugar-sweetened Drinks	34 (11, 94)	15 (0, 69)	<0.001	35 (11, 81)	54 (22, 94)	<0.001	9 (0, 60)	20 (0, 86)	0.07
Low calorie soft drinks	0.5 (0, 1)	0 (0, 0.5)	<0.001	0 (0, 0.5)	0.5 (0, 1)	<0.01	0 (0, 0.5)	0 (0, 0.5)	0.131
Tea/herbal infusions	0 (0, 18)	0 (0, 18)	<0.01	18 (0, 56)	0 (0, 18)	0.001	6 (0, 24)	0 (0, 6)	<0.001
Milk/chocolate milk	98 (32, 229)	41 (0, 134)	<0.001	98 (32, 229)	98 (28, 229)	0.44	56 (0, 134)	34 (0, 134)	0.23
Milkshakes/sherbets	0 (0, 0)	0 (0, 0)	<0.001	0 (0, 0)	0 (0, 0)	0.87	0 (0, 0)	0 (0, 0)	0.27
Coffee	96 (32, 225)	48 (8, 151)	<0.001	117 (32, 225)	96 (32, 225)	0.78	48 (5, 149)	48 (8, 151)	0.78
Alcoholic drinks	22 (0, 62)	44 (6, 170)	<0.001	22 (0, 22)	22 (22, 68)	<0.001	22 (0, 85)	83 (28, 257)	<0.001

All variables are presented as P50 (P25–P75). *p*-Values derived through the Mann-Whitney *U*-test for the skewed variables. ^a^ refers to comparisons between summer and winter for the total sample (males and females together); ^b^ refers to comparisons between males and females in winter; ^c^ refers to comparisons between males and females in summer.

The contribution of selected drinks to total energy intake is juxtaposed to their contribution to water intake (Table 4). It appears that coffee, milk/chocolate milk and alcoholic drinks are the main contributors to drink energy intake in winter and in summer, providing 79.5% and 69.5% of drink energy intake in winter and in summer. Solid foods provided, in winter and summer, respectively, approximately 78% and 74% of total energy intake, while drinks 22% and 26%. This may be further analyzed, considering categories of drinks. The main contributors to energy intake were milk/chocolate milk, coffee/coffee drinks and alcoholic drink provided, respectively, in winter 31.6%, 35.7% and 12.2% and in summer 23.3%, 22.2% and 24% of drink energy intake. Fruit juice, SSDs, tea/herbal infusions, and milkshakes/sherbets contributed less energy to daily intake (Table 4).

**Table 4:** Contribution of drinks in total water intake (TWI), total energy intake (TEI), drink water intake (DWI) and drink energy intake (DEI) during winter and summer.

Drinks	TWI (%)	DWI (%)	TEI (%)	DEI (%)
Winter				
Fruit juice 100%	3.3	11.8	1.7	9.2
Sugar-sweetened Drinks	4.7	15.4	0.5	2.5
Low calorie soft drinks	0.6	2.2	0.03	0.2
Tea/herbal infusions	2.6	9.9	1.4	7.1
Milk/chocolate milk	6.6	22.5	6.6	31.6
Milkshakes/sherbets	0.2	0.7	0.5	1.6
Coffee	8.6	30.0	7.9	35.7
Alcoholic drinks	2.4	8.3	2.6	12.2
Summer				
Fruit juice 100%	2.1	8.5	2.7	10.6
Sugar-sweetened Drinks	3.1	11.2	2.8	11.4
Low calorie soft drinks	0.8	3.1	0.04	0.2
Tea/herbal infusions	2.0	7.0	1.4	6.4
Milk/chocolate milk	4.0	14.8	5.0	23.3
Milkshakes/sherbets	0.2	0.7	0.4	1.8
Coffee	7.1	28.0	5.2	22.2
Alcoholic drinks	5.3	26.9	7.2	24.0

A more descriptive presentation of consumption of “SSDs” may be of interest because it reveals in more detail the contribution of various beverages. Consumption of SSDs consisted of fruit nectar with sugar (32%), soft drinks with sugar (63%) and energy/isotonic drinks (5%); consumption of ‘fruit juice 100%’ consisted of fresh fruit juice (50%), and from packed fruit juice 100% without sugar content (50%); the consumption of ‘tea/herbal infusions’ consisted of tea (69%), and other herbal infusions (31%), the consumption of ‘milk/chocolate milk’ consisted of milk (81%), chocolate milk (6%) and cocoa drink (13%); and the consumption of ‘alcoholic drinks’ consisted of beer (43%), wine (46%) and other types of alcoholic drinks (11%).

The correlation between the contribution of drinks to water intake and to energy in winter and summer was linear (Pearson’s *r* = 0.713, *p* < 0.001 and Spearman’s rho = 0.881, *p* < 0.001, respectively). To further understand consumer choices, according to whether they consume low or high drinks energy, data were classified in quartiles according to drink energy intake (Table 5). It was observed that people classified to the category of high drink energy intake, also had higher drink water intake WI (*p* < 0.001) but similar BMIs (*p* = 0.95, *p* = 0.92 in winter and summer, respectively) and energy expenditure from physical activity. When the values of winter quartiles were used as cut-offs to categorize the data collected in summer it was observed that the 30%, 21%, 22% and 27% of the participants was falling in these four categories. The higher values observed in the highest and lowest category signify different distribution in drink energy intake in winter than in summer and is in accordance to previous observations on patterns for total water intake in summer and in winter.

**Table 5:** Energy balance, intake and loss, ^a^ during winter, according to quartiles of energy intake from drinks during winter. Energy balance, intake and loss, ^a^ during summer, according to quartiles of energy intake from drinks during winter.

	Quartiles of Energy from Drinks (kcal/day) ^b^				*p* ^c^
	1st Quartile	2nd Quartile	3rd Quartile	4th Quartile	
	(<286)	(286 to 415)	(415 to 605)	(>605)	
*Winter, (%)*	*25*	*25*	*25*	*25*	
Energy Intake	1461 ± 473	1850 ± 558	2238 ± 655	2793 ± 1114	<0.001
Energy from foods	1192 (899, 1615)	1480 (1112, 1789)	1656 (1343, 2036)	1719 (1340, 2478)	<0.001
Energy expenditure	1804 ± 348	1889 ± 387	1885 ± 463	1862 ± 347	0.33
Estimated energy balance	−253 ± 574	81 ± 537	455 ± 734	1074 ± 1050	<0.001
BMI	24.6 ± 4.7	24.4 ± 4.3	24.7 ± 4.4	24.6 ± 4.3	0.95
Water from drinks	397 (316, 494)	578 (515, 677)	849 (734, 930)	1216 (1063, 1437)	<0.001
Water from drinking water	1257 ± 565	1424 ± 679	1401 ± 744	1442 ± 616	0.10
*Summer, (%)*	*30*	*21*	*22*	*27*	
Energy Intake	1427 ± 522	1647 ± 735	1992 ± 677	2625 ± 1065	<0.001
Energy from foods	1202 (942, 1527)	1148 (910, 1519)	1372 (1060, 1796)	1432 (1155, 1801)	<0.001
Energy expenditure	1772 ± 570	1810 ± 411	1901 ± 558	1859 ± 358	0.20
Estimated energy balance	−353 ± 754	−174 ± 757	75 ± 866	747 ± 1102	<0.001
BMI	24.6 ± 3.5	24.4 ± 4.4	24.7 ± 4.5	24.5 ± 3.7	0.92
Water from drinks	465 (311, 578)	744 (633, 922)	1078 (861, 1208)	1562 (1290, 2000)	<0.001
Water from drinking water	2118 ± 1120	2271 ± 1142	2270 ± 1349	2183 ± 978	0.67

^a^ Results are presented as as mean ± SD for the normally distributed variables (*i.e.*, energy intake, energy expenditure, energy balance, water intake, water from drinking water, water loss, water balance, BMI) and as P50 (P25–P75) for the skewed variables (*i.e.*, energy from foods, water from drinks, water from foods); ^b^ Quartiles of water balance were defined according to energy intake from drinks during winter; ^c^ *p*-Values derived through One Way Anova for the normally distributed variables and through Kruskal-Wallis H test for the skewed variables.

## 5. Discussion

### 5.1. Overview of Contribution of Drinks to Total Energy Intake

The first finding was that drinks contribute approximately 1/4 of the total energy intake of study population; drink energy intake was 479 ± 286 Kcal, *i.e.*, 23% of total energy intake in winter and 492 ± 499 Kcal, *i.e.*, 26.8% of total energy intake in summer in both sex. Total energy intake reported herein was compared with previously published estimations. Data from Greece are available from the ATTICA and EPIC study [18,25] and show similar total energy intake. There are no published data for contribution of drink energy intake to total energy intake from Greece. In other countries, contribution of drink energy intake to total energy intake may differ. For example, in the UK, Gibson and Shirreffs [26] reported that drink energy intake was 17.3% or 14.4% of total energy intake for men or women. In the National Health and Nutrition Examination Survey in the US, data from 1999–2002 show that drink energy intake was 22%, 17.8% and 14.5% of total energy intake in men 20–39 years, 40–59 years and ≥60 years, respectively, and 18.9%, 15.4% and 12.5% in women 20–39 years, 40–59 years and ≥60 years, respectively [27], also data from 2005–2010, reported that drink energy intake was 21.7% or 17% of total energy intake in adults 20–50 years or 51–70 years [28].

The second finding was that seasonality and sex affect drink energy intake and total energy intake; drink energy intake, as well as total energy intake and energy intake from solid foods, was higher in winter than in summer and in men than in women. There are no data in the literature on the effect of seasonality by sex to the contribution of drink energy intake to total energy intake.

The third finding, revealed when dividing the sample in quartiles according to drink energy intake, was that participants receiving higher drink energy intake also had higher total energy intake and energy intake from solid foods, similar energy expenditure due to physical activity and higher estimated energy balance. However, despite differences in energy intake, the BMIs of the participants were similar. It may be that BMIs were estimated inaccurately, because weight and height were self-reported. This method, although frequently used, is associated with bias because of difficulties with recall or underreporting of weight seeking social desirability [29]. Nevertheless, the association between drink energy intake and obesity may be complex; for example in a US population group, overweight and obese adults consumed more diet beverages than healthy-weight adults; moreover, they receive more energy from solid-food calories and total energy intake than overweight and obese adults who drink SSDs [30]. It must be noted that this is a cross-sectional study, therefore causal inference must not be made in any case and the relationship between BMI and energy intake from beverages is not to be highlighted herein. Nevertheless it is of interest to mention this finding as a result that deserves further investigation.

### 5.2. Energy Contribution of Selected Drinks

The fourth finding was that not all drinks contribute to total energy intake with the same manner. It appears that some, being consumed more frequently and in various forms and recipes, contribute more energy than others. The main contributors to drink energy intake, both in winter and summer were milk/chocolate milk, coffee/coffee drinks and alcoholic drinks. These add up to approximately 75% of drink energy intake. Coffee drinks, most of the times sweetened with sugar, caramel syrup and accompanied with whipped cream, are of high-energy content and are frequently consumed. Milk and other dairy drinks are also among the main contributors to drink energy intake. Fruit juice, SSDs, tea/herbal infusions sweetened with sugar or honey, milkshakes/sherbets and alcoholic drinks contributed less energy to daily intake. It was observed that SSDs and fruit juices contribute, respectively, in winter 0.5% and 1.7% and in summer 2.8% and 2.7% of total energy intake. The contribution of specific drinks to total energy intake is different in Greece than in other countries, although drink energy intake as well as drink energy intake contribution to total energy intake was similar. For example, NHANES study in U.S. revealed that soft-drinks/soda were the third of the highest ranked sources of energy among the subgroup of adults 19–50 years, providing 6.6% and alcoholic beverages provided the 5.4% of energy [31]. Another study among 9491 adults in U.S. indicated major contributors to caloric intake from drinks were alcohol (381 Kcal/day), sodas/colas (297 Kcal/day) and fruit drinks (234 Kcal/day) [13], while a nutrition survey among Canadians indicated that fruit juice and fruit drinks contribute 2.7% and soft drinks-regular contribute 2.6% of total daily energy [32]. In 2008–2009 alcoholic beverages provided 770 kJ/day (184 kcal/day) per capita, and caloric soft drinks (soda and juice drinks) 209 kJ (50 kcal/day) per capita; providing a concern in dominance of alcohol [33]. A recent study shows that alcoholic drinks in particular were a major contributor to evening and weekend peaks in beverage consumption [25]. Alcoholic drinks may be consumed for pleasure, but do not offer nutritional value and functional benefit while a gram of alcohol provides 7.1 Kcal.

The association between SSB consumption and obesity/weight gain remains a complex and controversial issue despite its great importance for clinical and public health issues. It has been the focus of many studies and of scientific reviews, some of which have been questioned for their scientific objectivity; it was observed that financial conflicts of interest, namely industry funding, may bias conclusions from systematic reviews on SSB consumption and weight gain or obesity [34,35].

Sex differences in patterns of energy intake from selected drink categories were identified in both seasons. In winter and in summer men had higher drink energy intake than women from SSDs and alcoholic drinks, while women had higher intake from sweetened tea and herbal infusions. This is in agreement with observations in populations from other countries. For example, according to data from the 1995 National Nutrition Survey by the NSW Centre in Australia, among adults, the highest consumers of SSDs were males [36]. There are no data in the literature for comparing drink consumption in summer and winter. Therefore, data reported herein are of particular interest and reflect lifestyle features in areas that have mild winter and hot summers.

### 5.3. Correlation of Water and Total Energy Intake

The fifth finding was that the correlation between drink energy intake and drink water intake was linear; moreover, higher water intake from foods and drinks was observed in the quartile of higher drink energy intake, which also had higher total energy intake and higher energy balance. This suggests that the selection of hydration source for each individual may be in accordance to the healthy/unhealthy dietary habits of this individual.

Though drinking water may not have the hedonic appeal and taste of caloric beverages, water is an important source of fluid for hydration because of wide availability, low cost and no caloric content; therefore water maybe a preferable choice for quenching thirst. It has been reported that replacing sweetened caloric drinks with drinking water was linked to a decrease in total energy intake [16].

The results of this study may be exploited in view of a number of limitations. One refers to the potential misreporting of dietary intake, frequently occurring when employing the FFQ methodology [37]. The FFQs estimate the usual food intake and require little time to complete, but many details of diet intake are not measured, mainly concerning the estimation of the size of the portion consumed. Recent research encourages the use of FFQ for the assessment of usual meal patterns, in addition to dietary diaries or recalls seems to be the best approach [38]. Another limitation regarding the FFQ analysis is that there are not detailed food-composition tables for the foods consumed in our country, so USDA tables and additionally the software enriched with selected Greek recipes. Additionally, regarding the self-reporting of the energy intake, there is evidence from validation studies that is underestimated by about 20%–25% in the dietary surveys [39]. Another limitation is that weight and height was self-reported, leading to inaccuracy when estimating BMI. Furthermore, it must be noted that this is a cross-sectional study and it is difficult to make causal inference; therefore, the relationship between BMI and energy intake from beverages is not to be highlighted in this study.

## 6. Conclusions

In conclusion, this study takes the importance of drinks to hydration as a starting point and further builds up, linking hydration with energy intake. Drinks hydrate but also contribute to total energy intake according to dietary habits and the energy content of the drink. In the population tested, regardless of seasonal and sex differences, drinks contributed approximately 1/4 of total energy intake, coffee, dairy and alcoholic drinks being the major contributors.
